# Caspase inhibition rescues F1Fo ATP synthase dysfunction-mediated dendritic spine elimination

**DOI:** 10.1038/s41598-020-74613-9

**Published:** 2020-10-16

**Authors:** Hao Chen, Jing Tian, Lan Guo, Heng Du

**Affiliations:** 1 Department of Biological Sciences, The University of Texas at Dallas, 800 west Campbell Rd, Richardson, TX 75080 USA; 2 Higuchi Biosciences Center, The University of Kansas, Lawrence, KS 66045 USA; 3 Department of Pharmacology & Toxicology, The University of Kansas, Lawrence, KS 66045 USA

**Keywords:** Cell biology, Molecular biology, Neuroscience

## Abstract

Dendritic spine injury underlies synaptic failure in many neurological disorders. Mounting evidence suggests a mitochondrial pathway of local nonapoptotic caspase signaling in mediating spine pruning. However, it remains unclear whether this caspase signaling plays a key role in spine loss when severe mitochondrial functional defects are present. The answer to this question is critical especially for some pathological states, in which mitochondrial deficits are prominent and difficult to fix. F1Fo ATP synthase is a pivotal mitochondrial enzyme and the dysfunction of this enzyme involves in diseases with spinopathy. Here, we inhibited F1Fo ATP synthase function in primary cultured hippocampal neurons by using non-lethal oligomycin A treatment. Oligomycin A induced mitochondrial defects including collapsed mitochondrial membrane potential, dissipated ATP production, and elevated reactive oxygen species (ROS) production. In addition, dendritic mitochondria underwent increased fragmentation and reduced positioning to dendritic spines along with increased caspase 3 cleavage in dendritic shaft and spines in response to oligomycin A. Concurring with these dendritic mitochondrial changes, oligomycin A-insulted neurons displayed spine loss and altered spine architecture. Such oligomycin A-mediated changes in dendritic spines were substantially prevented by the inhibition of caspase activation by using a pan-caspase inhibitor, quinolyl-valyl-O-methylaspartyl-[-2,6-difluorophenoxy]-methyl ketone (Q-VD-OPh). Of note, the administration of Q-VD-OPh showed no protective effect on oligomycin A-induced mitochondrial dysfunction. Our findings suggest a pivotal role of caspase 3 signaling in mediating spine injury and the modulation of caspase 3 activation may benefit neurons from spine loss in diseases, at least, in those with F1Fo ATP synthase defects.

## Introduction

Dendritic spines are dynamic bulbous protrusions on dendritic branches housing postsynaptic components^1^. Dendritic spine plasticity has a close relationship with brain function in physiology and pathology. Highly active during development to sculpt neural circuits^2^, dendritic spine turnover is relatively slow in developed brains for the storage of memory^3,4^. However, dendritic spine elimination become prominent at disease states such as brain aging and neurodegenerative disorders including Alzheimer’s disease (AD), leading to compromised learning capacity and impaired memory^5–9^. It is a well-documented notion that spine motility is regulated by synaptic activity^10–12^. In recent years, the role mitochondria in the control of spinogenesis and dendritic spine dynamics is increasingly recognized ^13–18^.

Mitochondria are crucial for neuronal activity and survival. In addition to their function in energy provision and local Ca^2+^ homeostasis maintenance, mitochondria-mediated pro- apoptotic signaling constitute the intrinsic pathway for programmed cell death^19^. Mitochondria are dynamic organelles and abundantly distributed in dendrites with physical proximity to dendritic spines^14,15^. Based on the spatial relationship between mitochondria and spines, it is proposed that mitochondria are important for spine structure and function, at least, through local ATP provision and Ca^2+^ regulation. This idea is strengthened by experimental observations^13–18^. Intriguingly, emerging evidence highlights a role of mitochondria-mediated non-lethal local caspase signaling in dendritic spine pruning^20–22^. However, the release of mitochondrial pro-apoptotic factors requires disruption of mitochondrial membrane structural integrity^19^, which leads to reduced mitochondrial OXPHOS efficacy and other functional defects. Therefore, the identification of the importance of nonapoptotic local caspase signaling in dendritic spine elimination has raised a critical scientific question about the major player in mitochondrial pathway of spine regulation. The answer to this question is critical especially for some pathological states, in which mitochondrial defects are prominent and difficult to fix.

F1Fo ATP synthase is a pivotal mitochondrial enzyme converting ADP to ATP through oxidative phosphorylation (OXPHOS)^23^. The relevance of F1Fo ATP synthase dysfunction to neurological disorders exhibiting dendritic spinopathy such as neuropathy, ataxia, and retinitis pigmentosa (NARP) ^24,25^, AD^26^ and many others^27^ suggests a link between F1Fo ATP synthase disruption and dendritic spine loss. Moreover, in our previous study, F1Fo ATP synthase dysfunction through downregulation of oligomycin sensitivity conferring protein (OSCP) induced severe postsynaptic activity defects in neurons^26^, further implicating the deleterious impact of F1Fo ATP synthase deregulation on spine function. In view of its influence on mitochondrial function, inhibition of F1Fo ATP synthase in neurons may offer us a research approach to determine the importance of caspase-dependent spine elimination pathway in response to mitochondrial functional deficits.

In this study, we induced F1Fo ATP synthase dysfunction by using oligomycin A. Short-term (30 min) treatment of oligomycin A did not affect neuronal viability; while oligomycin A-treated neurons underwent mitochondrial changes including decreased mitochondrial bioenergetics, enhanced mitochondrial superoxide production along with increased dendritic mitochondrial fragmentation and reduced distribution to spines. In addition, sublethal oligomycin A treatment induced nonapoptotic caspase 3 signaling activation in dendrites as demonstrated by Bax translocation onto dendritic mitochondria, cytochrome C release and caspase 3 cleavage. These changes concurred with dendritic spine retraction and spinogenesis suppression. Importantly, F1Fo ATP synthase dysfunction-related spine changes were prevented by the application of quinolyl-valyl-O-methylaspartyl-[-2,6-difluorophenoxy]- methyl ketone (Q-VD-OPh), a potent pan-caspase inhibitor^28^, although Q-VD-OPh was not protective against oligomycin A-mediated mitochondrial dysfunction. Our findings suggest that local caspase 3 activation plays a pivotal role in mediating spine injury triggered by F1Fo ATP synthase dysfunction. Modulation of caspase activation may benefit neurons from spine loss in diseases with F1Fo ATP synthase defects.

## Results

### Sublethal oligomycin A treatment dampens mitochondrial bioenergetics and arouses oxidative stress

**Figure 1:**
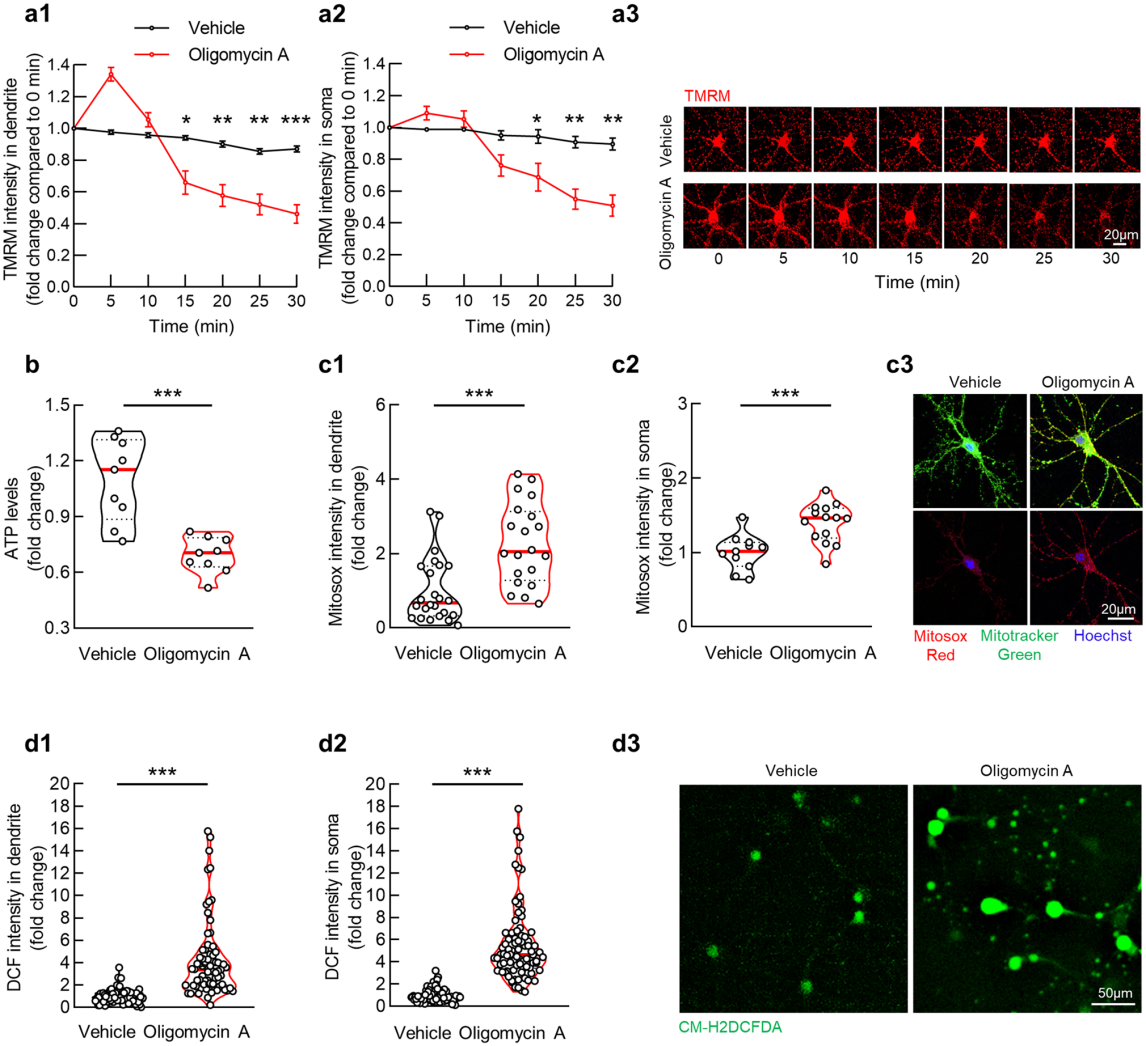
Oligomycin A treatment compromises mitochondrial function in cultured hippocampal neurons. (**a**) TMRM intensity indicated cultured hippocampal neuron dendritic (**a1**) and somatic (**a2**) mitochondrial membrane potential with vehicle or 1 µM oligomycin A treatment. Unpaired student’s *t*-test; *P < 0.05, ***P < 0.001. *n* = 5–10 cells or *n* = 15–20 dendritic segments per group, respectively. (**a3**) Representative live images of TMRM, images were recorded at time 0,5,10,15,20,25,30 min. Scale bar 50 µm. (**b**) Hippocampal neuronal ATP production with vehicle or 1 µM oligomycin A treatment. Unpaired student’s *t*-test; ***P < 0.001. *n* = 9 samples each group. (**c**) Mitosox Red intensity indicated cultured hippocampal neuron dendritic (**c1**) and soma (**c2**) Mitochondrial ROS level with vehicle or 1 µM oligomycin A treatment. Unpaired student’s *t*-test; ***P < 0.001. *n* = 12–15 cells or *n* = 20 dendritic segments per group, respectively. (**c3**) Representative images of Mitosox Red staining. Mitochondria are labeled with Mitotracker green. Hoechst was applied for nucleus staining. Scale bar 20 µm. (**d**) CM- H2DCFDA intensity indicted cultured hippocampal neuron dendritic (**d1**) and soma (**d2**) intra-neuronal ROS level with vehicle or 1 µM oligomycin A treatment. Unpaired student’s *t*-test; ***P < 0.001. *n* = 80–100 cells or *n* = 45 dendritic segments per group, respectively. (**d3**) Representative images of CM- H2DCFDA (green). Scale bar 50 µm.

To induce F1Fo ATP synthase dysfunction, we exposed primary cultured hippocampal neurons to oligomycin A, which inhibits F1Fo ATP synthase proton flow through its binding with the c subunit^29^. Characterization of the cell culture showed a high purity of neural cells (Supplementary Fig. S1a,b). The impact of oligomycin A on neuronal survival was determined using Calcein AM staining, a reliable fluorescent dye-based cell viability assay^30^. Thirty minutes’ treatment of oligomycin A at concentrations up to 10 µM demonstrated no effect on neuronal survival (Supplementary Fig. S2a,b). Based on its potent inhibitory effect^31^, we therefore used oligomycin A at a sublethal low concentration (1 µM) for mitochondrial bioenergetics assays. We first determined the effect of oligomycin A on mitochondrial membrane potential (mΔΨ) by measuring the kinetic change of tetramethylrhodamine (TMRM) intensity^32^ in response to oligomycin A insult. After a transient increase in their membrane potential, oligomycin A-insulted mitochondria in dendrites (Fig. 1a1,a3) and in soma (Fig. 1a2,a3) exhibited remarkably decreased TMRM intensity at the end of the 30 min’ observation. Such a delayed mΔΨ-collapsing effect of oligomycin A treatment is in agreement with previous reports^31^. Mitochondrial membrane potential is the driving force for ATP production via F1Fo ATP synthase^23^. Echoing mitochondrial depolarization, oligomycin A-treated neurons showed decreased ATP content in comparison with their vehicle-treated control (Fig. 1b). The results indicate suppressed mitochondrial bioenergetics due to F1Fo ATP dysfunction.

Although physiological mitochondrial depolarization favors less mitochondrial reactive oxygen species (ROS) production, damaged mitochondrial respiration and OXPHOS promotes free radicals generation^33,34^. Consistent with previous studies^35^, F1Fo ATP synthase inhibition in cultured hippocampal neurons induced significantly increased superoxide levels in dendritic (Fig. 1c1,c3) and somatic (Fig. 1c2,c3) mitochondria as demonstrated by augmented intensity of MitoSox Red, a specific mitochondrial superoxide indicator^26^. The results support oligomycin A neurotoxicity in mediating mitochondrial ROS burst. Consistent with the notion that mitochondrial ROS overproduction breaks intra-neuronal Redox balance^26,36^, oligomycin A-treated neurons had increased ROS levels in their dendrites (Fig. 1d1,d3) and soma (Fig. 1d2,d3) as determined by means of CM-H2DCFDA staining^37^. These data in together suggest the deleterious impact of oligomycin A at a sublethal concentration on mitochondrial function.

### Sublethal oligomycin A treatment increases dendritic mitochondrial fragmentation and reduces mitochondrial distribution to spines

**Figure 2:**
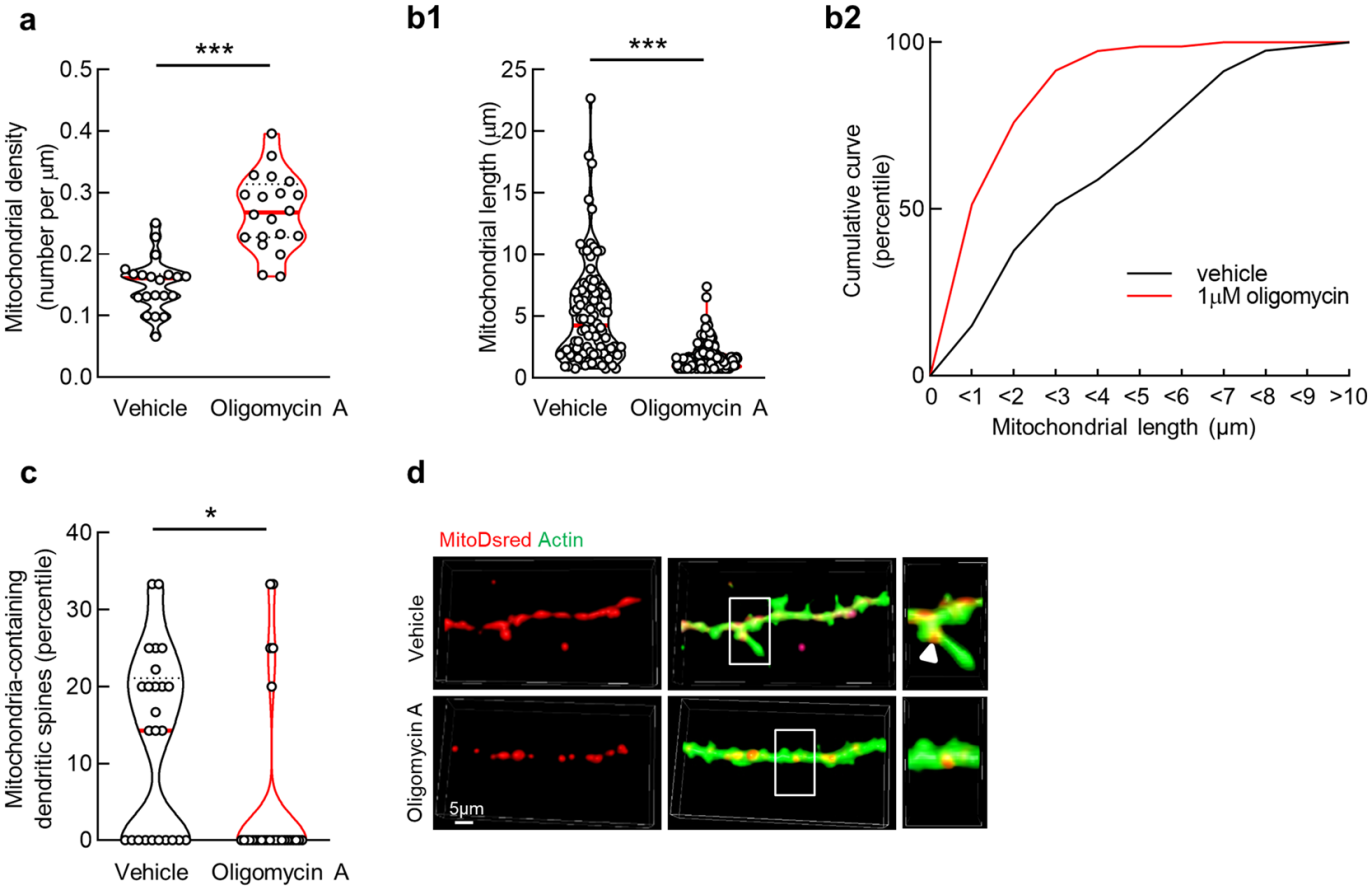
Oligomycin A treatment upregulates dendritic mitochondrial fragmentation and reduces spinal mitochondrial distribution in cultured hippocampal neuron. (**a**) Dendritic mitochondrial density of cultured hippocampal neurons with vehicle or 1 µM oligomycin A treatment. Unpaired student’s *t*-test; ***P < 0.001. *n* = 20 dendritic segments. (**b1**) Dendritic mitochondrial length of cultured hippocampal neurons with vehicle or 1 µM oligomycin A treatment. Unpaired student’s *t*-test; ***P < 0.001. *n* = 92 and 132 mitochondria, respectively. (**b2**) The corresponding cumulative curve of (**b1**). (**c**) The ratio of mitochondria-containing dendritic spines in cultured hippocampal neurons with vehicle or 1 µM oligomycin A treatment. Unpaired student’s *t*-test; *P < 0.05. *n* = 25–30 dendritic segments. (**d**) Representative images of dendritic spines (green) and mitochondria (red). Scale bar 5 µm.

Mitochondria are highly dynamic organelles and mitochondrial distribution to dendritic spines is pivotal for spinogenesis and spine function^14,15^. To fully depict the effect of F1Fo ATP synthase inhibition on dendritic mitochondria, we examined mitochondrial dynamics. Mitochondria were determined by particles expressing MitoDsRed^14^. When compared with their counterparts in vehicle-treated neurons, oligomycin A-exposed dendritic mitochondria demonstrated increased numbers (Fig. 2a,d) and reduced length (Fig. 2b1,d) with a leftward shift in the cumulative length distribution (Fig. 2b2), implicating active mitochondrial fragmentation in response to oligomycin A. Moreover, with the co-staining of ActinGreen 488 ReadyProbes to visualize dendritic protrusions^14^, we found a substantial decrease in the percentage of mitochondria-containing dendritic spines after oligomycin A treatment (Fig. 2c,d). These data suggest that F1Fo ATP synthase inhibition by oligomycin A alters dendritic mitochondrial dynamics and suppresses mitochondrial migration to dendritic spines. Of not, in comparison with their dendritic siblings, somatic mitochondria showed similar changes in mitochondrial density (Supplementary Fig. S3a,d), length (Supplementary Fig. S3b,d), and area (Supplementary Fig. S3c,d) in response to oligomycin A exposure, suggesting a pan-effect of oligomycin A on the dynamics of mitochondria in different neuronal sub-compartments.

### Sublethal oligomycin A treatment triggers caspase 3 activation in dendrites

**Figure 3:**
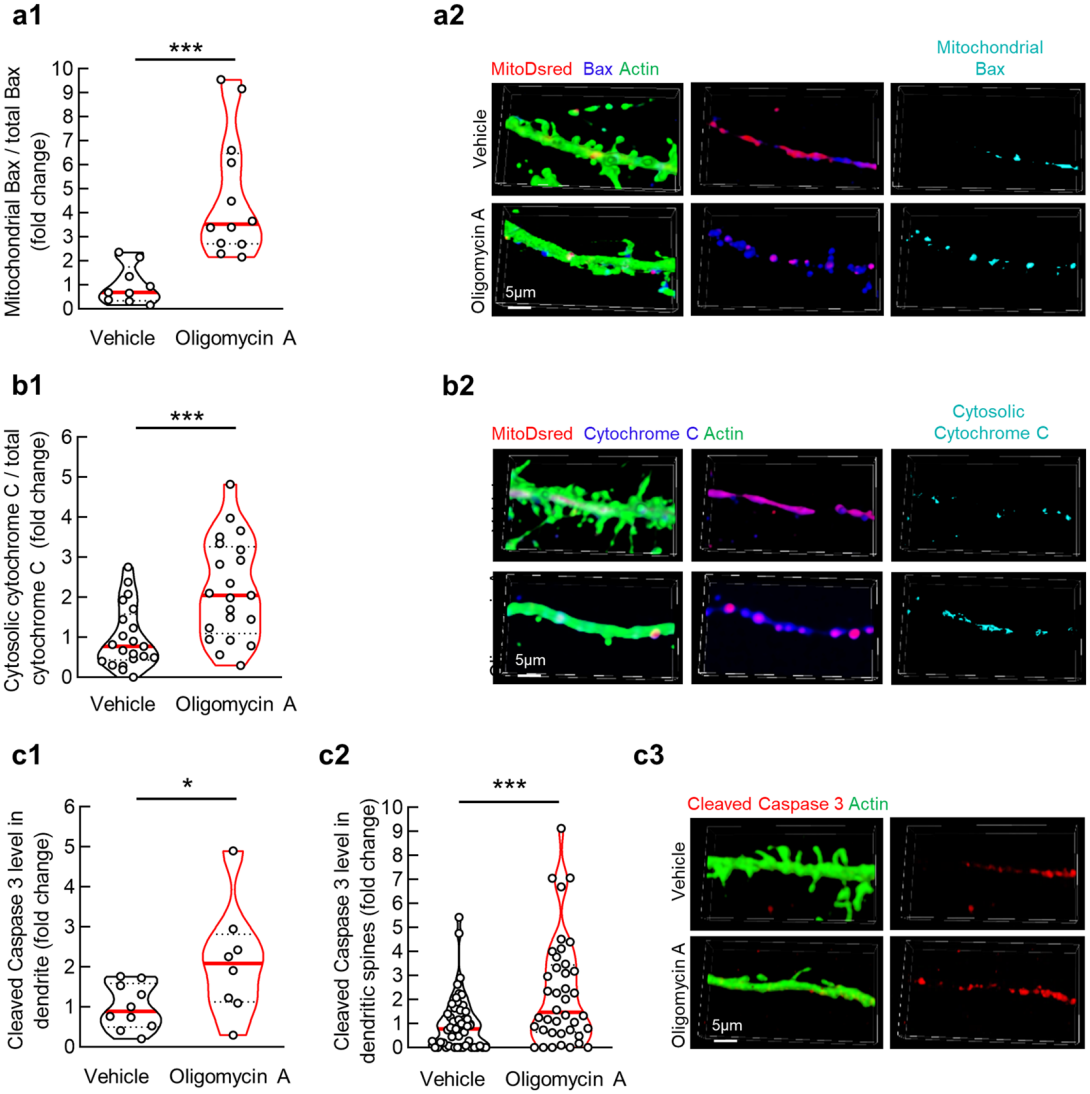
Oligomycin A treatment activates dendritic local apoptotic signaling in cultured hippocampal neurons. (**a1**) Dendritic mitochondria translocated Bax levels in cultured hippocampal neurons with vehicle or 1 µM oligomycin A treatment. Unpaired student’s t-test; ***P < 0.001. n = 9–12 dendritic segments. (**a2**) Representative images of mitochondria (red), Bax (blue) and dendrites (green). Scale bar 5 µm. (**b1**) Dendritic Cytochrome C release levels in cultured hippocampal neurons with vehicle or 1 µM oligomycin A treatment. Unpaired student’s t-test; ***P < 0.001. n = 21 dendritic segments. (**b2**) Representative images of mitochondria(red), Cytochrome C (blue) and dendrites (green). Scale bar 5 µm. (**c1**) Cleaved Caspase levels in cultured hippocampal neuron dendrite (**c1**) or dendritic spine (**c2**) with vehicle or 1 µM oligomycin A treatment. Unpaired student’s t-test; *P < 0.05. n = 10 dendritic segments. (**c3**) Representative images of Cleaved Caspase 3 (red) and dendrites (green). Scale bar 5 µm.

Given the close association between mitochondrial damages and the release of pro- apoptotic factors from mitochondria^38^, we sought to determine whether oligomycin A-induced F1FO ATP synthase dysfunction could mediate mitochondrial pathway of caspase 3 activation in dendrites. Neurons with MitoDsRed-labeled mitochondria were subjected to the vehicle or oligomycin A treatment, followed by the staining of Bax. As compared with dendritic mitochondria in the vehicle group, oligomycin A-treated dendritic mitochondria exhibited remarkably enhanced Bax translocation (Fig. 3a1,a2). Bax translocation onto mitochondria triggers the release of cytochrome C. In comparison to the containment of cytochrome C in mitochondria in the vehicle-treated neurons, oligomycin A-exposed neurons showed increased cytochrome C staining outside their dendritic mitochondria (Fig. 3b1,b2). The release of Cytochrome C from mitochondria is a key triggering factor for caspase 3 activation^19^. Further immunofluorescent staining for cleaved caspase 3, the indicator of caspase 3 activation^19^ showed an augmentation in caspase 3 cleavage in dendritic shafts (Fig. 3c1,c3) and within dendritic spines (Fig. 3c2,c3). The promoting effect of oligomycin A on caspase 3 was further confirmed by immunoblotting (Supplementary Fig. S4a,b). Therefore, oligomycin A at the tested condition activates caspase 3 signaling in dendrites, at least in part, through a mitochondrial pathway without inducing neuronal death (Supplementary Fig. S2a,b).

### Caspase inhibition protects spine density and architecture from oligomycin A toxicity

**Figure 4:**
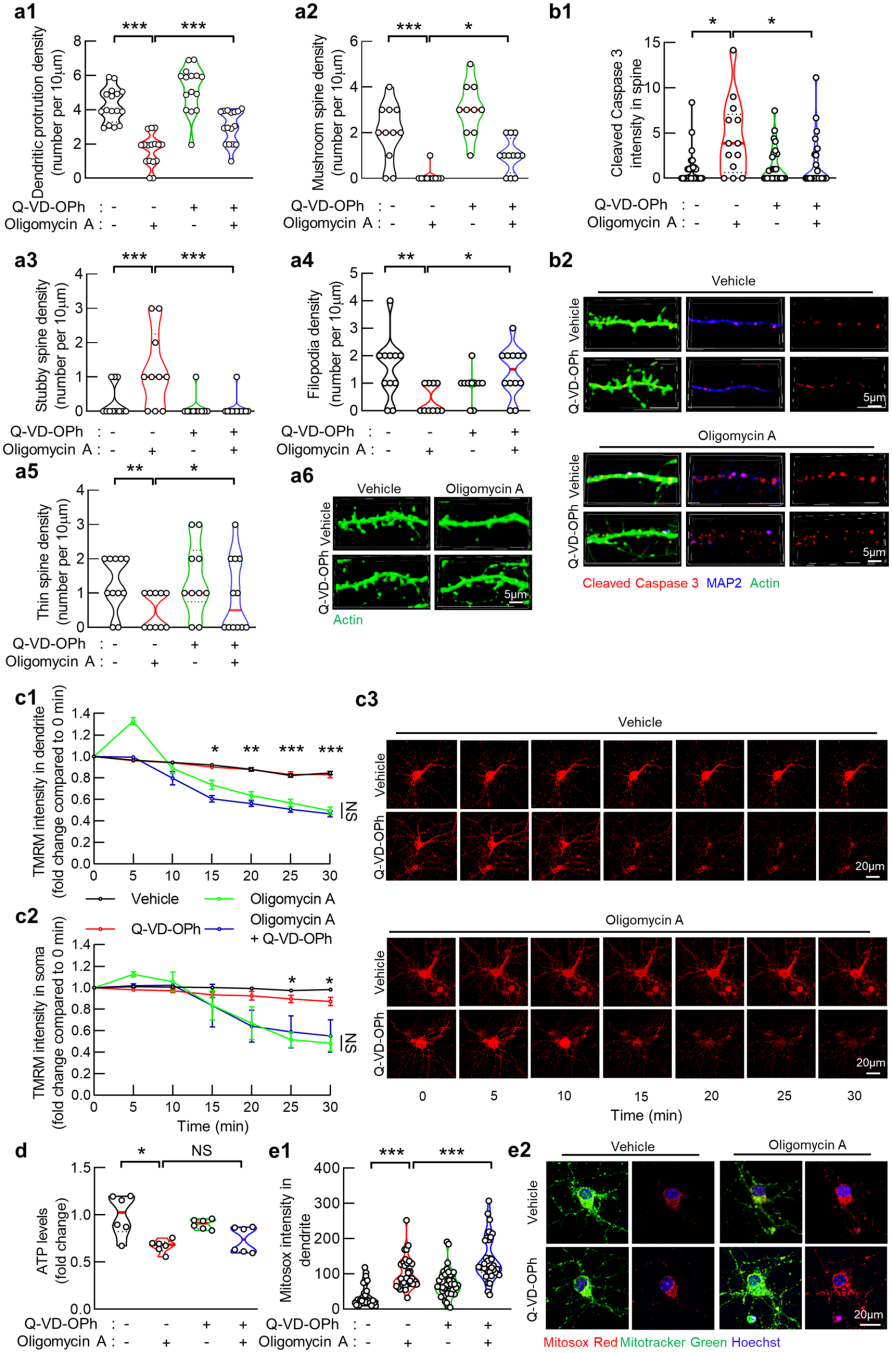
Caspase 3 inhibition protects spine density and architecture in oligomycin A treated neurons. (**a**) Attenuated oligomycin A-induced dendritic protrusion reduction in 1 µM Q-VD-OPh treated hippocampal neurons. (**a1**) Dendritic protrusion of vehicle**,** 1 µM Q-VD-OPh and/or 1 µM oligomycin A treated hippocampal neurons. (**a2**–**a5**) Density of multiple types of spine in vehicle**,** 1 µM Q-VD-OPh and/or 1 µM oligomycin A treated hippocampal neurons (**a2**) Mushroom spine, (**a3**) Stubby spine, (**a4**) Filopodia, (**a5**) Thin spine. Two-way ANOVA followed by Bonferroni post hoc analysis; *P < 0.05, **P < 0.01 and ***P < 0.001. *n* = 10–15 dendritic segments. (**a6**) Representative images of dendritic protrusions (green). Scale bar 5 µm. (**b1**) Cleaved Caspase 3 expression levels in dendritic protrusions of vehicle, 1 µM Q-VD-OPh and/or 1 µM oligomycin A treated hippocampal neurons. Two-way ANOVA followed by Bonferroni post hoc analysis; *P < 0.05. *n* = 15–30 dendritic protrusions. (**b2**) Representative images of Cleaved Caspase 3 (red), dendritic protrusions (green) and MAP2 (blue). Scale bar 5 µm. (**c**) Mitochondrial membrane potential of vehicle**,** 1 µM Q-VD-OPh and/or 1 µM oligomycin A treated hippocampal neuron dendritic (**c1**) and somatic (**c2**) mitochondria in the indicated treated hippocampal neurons. Two-way ANOVA followed by Bonferroni post hoc analysis; *P < 0.05, *NS* not significant,* n* = 35–40 dendritic segments, respectively. (**c3**) Representative images of TMRM. Images were recorded at time 0, 5, 10, 15, 20, 25, 30 min. Scale bar 50 µm. (**d**) Hippocampal neuronal ATP production under vehicle**,** 1 µM Q-VD-OPh and/or 1 µM oligomycin A treated. Two-way ANOVA followed by Bonferroni post hoc analysis; NS, not significant, *P < 0.05. *n* = 6 samples. (**e1**) Hippocampal dendritic mitochondrial ROS levels of vehicle**,** 1 µM Q-VD-OPh and/or 1 µM oligomycin A treated neuron cultures. Two-way ANOVA followed by Bonferroni post hoc analysis; NS, not significant, ***P < 0.001*, n* = 20–30 dendritic segments. (**e2**) Representative images of Mitosox Red staining. Mitochondria are labeled with Mitotracker green. Hoechst was applied for nucleus staining. Scale bar 20 µm.

To determine the impact of oligomycin A-mediated F1Fo ATP synthase deregulation on dendritic spines, we compared dendritic spine density and architecture between the vehicle- and oligomycin A-treated hippocampal neurons. In addition to decreased dendritic spine density (Fig. 4a1,a6), oligomycin A-insulted neurons exhibited a reduction in the density of mushroom type spines (Fig. 4a2,a6) with increased spines of stubby type (Fig. 4a3,a6). Moreover, when compared with the vehicle treatment, oligomycin A induced loss of filopodia (Fig. 4a4,a6) and thin spines (Fig. 4a5,a6). In view of the close relationship between spine morphological characteristics and function^39^, our findings support the deleterious impact of oligomycin A-mediated F1Fo ATP synthase deregulation on dendritic spine structure and function.

To dissect the contribution of caspase 3 activation to dendritic spine pruning from mitochondrial functional defects in oligomycin-A insulted hippocampal neurons, we blocked caspase activation by using quinolyl-valyl-O-methylaspartyl-[-2,6-difluorophenoxy]-methyl ketone (Q-VD-OPh), a potent pan-caspase inhibitor^28^. With the labeling of MAP-2 to identify the dendrites and actingreen to visualize dendritic spines, the addition of Q-VD-OPh significantly reduced cleaved caspase 3 intensity within dendritic spines in oligomycin A-treated neurons (Fig. 4b1,b2). Intriguingly, Q-VD-OPh per se had minimal effect on dendritic spines as compared with the vehicle treatment (Fig. 4a1,a6). However, in contrast with oligomycin A alone, the co-incubation of Q-VD-OPh substantially preserved the densities of total dendritic protrusions (Fig. 4a1,a6), mushroom spines (Fig. 4a2,a6), filopodia (Fig. 4a4,a6) and thin spines (Fig. 4a5,a6) with decreased density of stubby spines (Fig. 4a3,a6) from oligomycin A toxicity. Of note, the administration of Q-VD-OPh showed no protective effect on dendritic mitochondrial membrane potential (Fig. 4c1-3), ATP content (Fig. 4d) or dendritic mitochondrial MitoSox Red intensity (Fig. 4e1,e2) in oligomycin A-treated neurons, although Q-VD-OPh alone did not exhibit mitochondrial toxicity (Fig. 4c–e). The results indicate the effect of Q-VD-OPh in preventing caspase activation without correcting dendritic mitochondrial defects in the testing condition. Therefore, our findings support a critical role of nonapoptotic local caspase activation in eliminating spines and suppressing spinogenesis in response to oligomycin A-mediated F1Fo ATP synthase dysfunction.

## Discussion

Active spine turnover is extremely crucial for shaping neural circuits in the developing brain^40^. However, excess spine elimination underlies synaptic failure in neurological disorders^5–9^. Therefore, to delineate mechanisms of spine pruning in pathological states is of paramount importance for the understanding of the pathogenesis of diseases and the development of therapeutic avenues. Previous studies have established a firm association between synaptic activity and dendritic spine regulation in cells and animals^10–12^. Long-term depression (LTD) is believed to promote dendritic spine retraction through repeated activation of N-methyl-D-aspartate receptor (NMDAR)^12^. Interestingly, the finding that caspase 3 activation contributes to activity-dependent spine shrinkage together with the NMDA- mediated mitochondrial toxicity in neurons^41^ seem to set up a link with mitochondrial pathway for spine regulation^12^. Indeed, the importance of mitochondria-mediated local caspase signaling in the control of spine density and architecture has been repeatedly identified^20–22^. The activation of mitochondria-related caspase signaling is the downstream of mitochondrial apoptotic morphological changes^19^. In view of the critical role of mitochondrial function in supporting spine growth and stability^42^, it is natural to propose a dual effect of mitochondrial stress on dendritic spine turnover through mitochondrial functional defects and mitochondrial pathway of local caspase activation in diseases. Intriguingly, recent studies suggested neuronal glycolysis as an alternative energy source in lieu of mitochondrial OXPHOS to support, at least, basal pre-^43^ and post-synaptic^44^ functions when mitochondrial ATP provision is not available or sufficient. In this context, correction of mitochondrial defects without manipulating caspase signaling may not be sufficient to rescue neurons from losing their spines in pathological states. Indeed, in this study we stressed mitochondria with oligomycin A-mediated F1Fo ATP synthase inhibition and observed spine injury along with dendritic mitochondrial defects. Aside from the fact that oligomycin A-induced mitochondrial dysfunction is the triggering factor for spine damages, the inhibition of caspase activation by using Q-VD-OPh demonstrated a substantial protection on spines against oligomycin A toxicity. Importantly, the administration of Q-VD- OPh demonstrated little or no protection on mitochondria. These findings support our hypothesis that, the nonapoptotic local caspase signaling, once activated, plays a major role in eliminating dendritic spines. Our findings are not an underestimation of the contribution of mitochondrial functional defects such as lowered ATP production, increased ROS generation and perturbed Ca^2+^ retention to spine loss. But they bring to our attention the effect of anti-local caspase activation in protecting spines. Of note, synapse trimming via microglial engulfment is another determined mechanism of spine elimination^45,46^. Emerging evidence suggests that local caspase signaling promotes synaptic changes that are recognized by activated microglia for further synapse pruning^47^. Therefore, the use of caspase inhibitors may supplement our current strategy to prevent spinopathy in neurological diseases, at least, in those with F1Fo ATP synthase deregulation such as brain aging^48^ and AD^26^.

Another important finding of our study is the deleterious impact of F1Fo ATP synthase dysfunction on dendritic mitochondrial dynamics and distribution. Mitochondria are dynamic organelles. Mitochondrial morphological control and appropriate positioning are important for neuronal activity and spine remodeling^14,49^. Although the detailed mechanisms of dendritic mitochondrial fragmentation and redistribution in response to F1Fo ATP synthase inhibition require further investigation, we attributed such changes to F1Fo ATP synthase dysfunction- related ATP deficiency, ROS overproduction and mitochondrial Ca^2+^ deregulation, which are determined factors affecting mitochondrial dynamics and motility^50^. Notably, mitochondrial fragmentation favors cytochrome C release from mitochondria^51^, which is in concordance with enhanced local caspase signaling due to F1Fo ATP synthase inhibition. Moreover, dendritic mitochondria demonstrate increased fission in response to activity-dependent spine remodeling^14^. In this scenario, cytochrome C release through activity-induced dendritic mitochondrial fission may be a critical physiological mechanism of spine elimination in the developing brain. This hypothesis will be addressed in our future study.

Intriguingly, a previous study showed a protective effect of F1Fo ATP synthase inhibition against excitatory neuronal death^52^. According to the hypothesis of mitohormesis^53^, mitochondrial stress may paradoxically confer cells resistance to lethal stimuli. In this regard, we cannot rule out the possibility that neurons actually benefit from F1Fo ATP synthase inhibition-instigated dendritic spine elimination in diseases. The crosstalk between spine plasticity and synaptic activity is supported by previous studies^12,54,55^. In this scenario, the potential benefits of mitochondrial malfunction, at least, to some extent should be acknowledged for treating neurological disorders by targeting mitochondria.

In summary, our findings support a pivotal role of mitochondrial pathway of local caspase signaling in mediating spine loss, especially in stress conditions with F1Fo ATP synthase dysfunction. It should be noted that our current study was performed on an in vitro experimental system with acute F1Fo ATP synthase modulation. Our findings need further in vivo studies with a more pathophysiologically relevant F1Fo ATP synthase manipulation, which form groundwork for our future investigation. Another critical issue is related to the contribution of local mitochondrial dysfunction-induced dendritic spine defects. Although we found that dendritic mitochondria were relatively vulnerable to oligomycin A insult in comparison with their somatic siblings, the treatment of oligomycin A exhibited non-selective effect on neuronal mitochondria in different sub-compartments. In this scenario, we cannot exclude a potential impact of the dysfunction of non-dendritic mitochondria on dendritic spine architecture. It is possible as somatic and pre-synaptic mitochondrial deficits may contribute to impaired synaptic transmission and activity, which indirectly affects dendritic spine plasticity. Therefore, in our future mechanistic study, we will induce mitochondrial dysfunction by employing optogenetic approaches to target selective groups of mitochondria in the dendrites. In regardless of these aforementioned limitations, this study fosters us a better understanding of a mitochondrial pathway of spinopathy and also sheds light to the use of caspase inhibitor as a therapeutic approach for treating neurological disorders.

## Materials and methods

### Mice usage, primary neuron culture and treatments

Animal studies were approved and performed under the guidelines of the Institutional Animal Care and Use Committee (IACUC) at the University of Texas at Dallas (UTD) and National Institutes of Health (NIH) under the protocol 13-01. C57BL/6 J mice were originally purchased from Jackson Lab and bred as needed. Mouse primary hippocampal neurons were cultured as previously described^26^. Day 0 pups’ brain were dissected in cold Dulbecco’s Modified Eagle’s Medium (DMEM, Sigma-Aldrich). Cells in mouse hippocampi were dissociated with 0.05% Trypsin at 37 °C for 25 min followed by 10 times trituration. Cells were then passed through 40 μm mesh cell strainer (Corning) and centrifuged for 5 min at 250 g. The pellet was gently resuspended in neuron culture medium (Neurobasal A with 2% B27 supplement, 0.5 mM l-glutamine, 1 µM 5-Fluoro-2′-deoxyuridine(FdU, Sigma-Aldrich F0503) ) and seeded on poly-D-lysine (Sigma-Aldrich) coated culture plates (Corning) or Lab-Tek chamber slides (Nunc, 177,445) with an appropriate density. Neurons were cultured to 10–13 days in vitro (DIV) and exposed to oligomycin A (Sigma-Aldrich) at different concentrations including 0, 1 and 10 μM for 30 min for cell viability measurement. For other experiments, the hippocampal neurons were treated with 1 μM oligomycin for 30 min. For caspase inhibition experiments, the hippocampal neurons were pre-treated with 1  μM quinolyl-valyl-O-methylaspartyl-[-2,6-difluorophenoxy]-methyl ketone (Q-VD-OPh, Abcam, ab141421) for 5 min before the addition of oligomycin A and followed by 30 min incubation.

### Lentivirus production

Mitochondria targeted DsRed (Clontech, Mountain View, CA, USA) was inserted into lentivirus vector with human polyubiquitin promoter-C (Addgene, Cambridge, MA, USA). HEK293T cells (ATCC, CRL-3216; RRID: CVCL_0063) were cultured in cell culture medium (DMEM + 10% FBS), one day before transfection, HEK293T cells were sub-cultured into 10 cm dish (Corning) at a density of 6 × 10^6^ cells, after 16 h, the cells were transiently co-transfected with packaging vector psPAX2 (Addgene) and envelope vector pMD2.G (Addgene) by using the standard calcium phosphate precipitation method^14^. At 24 h post-transfection, the medium was replaced with fresh DMEM and lentivirus-containing medium was harvested 24 h later. The virus supernatant was then filtered using a 0.45 μm PVDF filter (Millipore) to remove cell debris, concentrated by ultracentrifugation (25,000 rpm at 4 °C for 2 h), resuspended in neuron culture medium and stored at − 80 °C until use.

### Cell viability assay

Neuron viability was examined by Calcein AM staining^26^. Primary cultured hippocampal neurons with a density of 2 × 10^4^ per well on Lab-Tek chamber slides (Nunc, 177445) were incubated with 1 mM Calcein AM (Life Technologies, C3100MP) and 20 nM Hoechst (Thermo Fisher Scientific, 33342) for 20 min after indicated oligomycin A treatments. Treated neurons were washed with pre-warmed neuron culture medium 20 min after the staining. Images were collected on a Nikon inverted microscope with an on-stage incubator (5% CO_2_, 37 °C). The intensity of Calcein AM was subsequently analyzed by using Nikon NIS Advanced Research Elements 4.13.00 (https://www.microscope.healthcare.nikon.com/products/software/nis-elements/nis-elements-advanced-research).

### Mitochondrial membrane potential assay

Mitochondrial membrane potential was measured using Tetramethylrhodamine, methyl-ester (TMRM) as previously described^26^.Primary cultured hippocampal neurons with a density of 2 × 10^4^ per well on Lab-Tek chamber slides (Nunc, 177445) were incubated with 20 nM TMRM (Sigma-Aldrich, T5428) in neuron culture medium for 30 min in an incubator (5% CO_2_, 37 °C) before indicated treatments. Then neurons were followed by live imaging for up to 30 mins' recording time with a 5 min interval. Images were collected on a Nikon inverted microscope with an on-stage incubator (5% CO_2_, 37 °C). The intensity of TMRM was subsequently analyzed by using Nikon NIS Advanced Research Elements 4.13.00.

### Superoxide assay

Mitochondrial superoxide was determined by using Mitosox Red (Thermo Fisher Scientific, M36008). Primary cultured hippocampal neurons with a density of 2 × 10^4^ per well on Lab-Tek chamber slides (Nunc, 177,445) were pre-incubated with 2.5 µM Mitosox Red and 400 nM MitoTracker Green or 5 µM DCF for 20 min in an incubator (5% CO_2_, 37 °C) followed by a wash with pre-warmed Neurobasal A medium. Pre-stained neurons were treated with Oligomycin A as indicated in the previous section. 20 nM Hoechst (Thermo Fisher Scientific, 33342) was used to label the nucleus in live cells. The images of Mitosox Red were collected on a Nikon confocal microscope with on-stage incubator (5% CO_2_, 37 °C). The intensity was subsequently analyzed by using Nikon NIS Advanced Research Elements 4.13.00. Intra-neuronal superoxide was determined by CM-H2DCFDA (DCF, Thermo Fisher Scientific, C6827) staining as previously described^14,26^. Images of DCF staining were collected on a Nikon inverted microscope. The intensity was subsequently analyzed by using Addgene.

### ATP measurement

Cultured hippocampal neurons (2 × 10^5^ cells) ATP level with indicated treatments were analyzed by using ATP luminescent assay kit (Abcam Cambridge, MA, USA) following the manufacturer’s instructions.

### Immunoblotting analysis

Oligomycin A treated 2 × 10^5^ hippocampal neurons were collected in 1 × LDS buffer (50 mM Tris- HCl pH 6.8, 2% sodium dodecyl sulfate, 10% glycerol, 1% beta-mercaptoethanol, 12.5 mM EDTA and 0.02% bromophenol blue).Proteins were separated using NuPAGE 12% Bis–Tris Protein Gels (Invitrogen) and then transferred onto polyvinylidene difluoride membranes (PVDF, Bio- Rad Laboratories). After blocking in 5% w/v non-fat dry milk for 1 h at room temperature, membranes were probed with the following primary antibodies overnight at 4 °C^26^: rabbit anti-Total Caspase 3 (Cell Signaling Technology, #9662, 1:3000), rabbit anti-Cleaved Caspase 3 (Cell Signaling Technology, #9664, 1:1000). This was followed by probing with corresponding secondary antibodies for 1 h at room temperature: horseradish peroxidase-conjugated goat anti-rabbit IgG (H + L) (Invitrogen, 656120, 1:6000). Images were collected using Bio-Rad Chemidoc Imaging System. Bands were quantified using Image J (National Institutes of Health, NIH, https://imagej.nih.gov/ij/links.html).

### Immunocytochemistry

Immunocytochemistry staining was performed based on a published protocol^26^. Pretreated hippocampal neurons at a density of 2 × 10^4^ per well on Lab-Tek chamber slides (Nunc, 177445) were fixed in 4% paraformaldehyde (PFA) for 30 min then blocked with 5% Bovine Serum Albumin (BSA, Sigma-Aldrich) and 0.3% Triton-X-100 for 1 h at room temperature. Neurons were probed with proper combinations of the following primary antibodies overnight at 4 °C: mouse anti-Cytochrome C (Cell Signaling Technology, #12963, 1:600), rabbit anti-Bax (N20) (Santa Cruz, SC-493, 1:600), rabbit anti-Cleaved Caspase 3 (Cell Signaling Technology, #9664, 1:200) and mouse anti-MAP2 (Sigma-Aldrich, #M4403,1:600). Goat anti-mouse IgG conjugated with Alexa 647 (Cell Signaling Technology, #4410, 1:600), goat anti- rabbit IgG conjugated with Alexa 647 (Invitrogen, A21244, 1:600), goat anti-rabbit IgG conjugated with Alexa 594 (Invitrogen, A11037, 1:400) were used as fluorescent secondary antibodies. ActinGreen 488 ReadyProbes Reagent (R37110; Life Technologies, 1:400) was used to visualize dendritic protrusions. Dendritic segments between 70 and 100 μm from the soma were used for analysis. Images were collected under a Nikon confocal microscope followed by three-dimensional reconstruction using Nikon NIS Advanced Research Elements 4.13.00. The intensity of stained proteins were measured by threshold function of Nikon NIS Advanced Research Elements 4.13.00, the protein intensity of each layer was measured and was summed up to get the total intensity of the measured proteins. The ratio of Bax intensity in mitochondria to total Bax intensity in dendrites was used to stand for Bax translocation levels onto mitochondria and the ratio of cytosolic Cytochrome C intensity to total Cytochrome C intensity in dendrites was used to stand for Cytochome C releasing levels. For neuron purity determination, neurons were probed with proper combinations of the following primary antibodies overnight at 4 °C: goat anti-Iba1 (Abcam, ab5076, 1:600), rabbit anti-GFAP (Proteintech, 16825-1-AP, 1:600) and mouse anti-MAP2 (Sigma-Aldrich, #M4403, 1:600). After washing with PBS, the slices or neurons were then probed with appropriate cross-adsorbed secondary antibodies conjugated to Alexa Fluor 488 (Thermo Fisher Scientific, 1:500), Alexa Fluor 594 (Thermo Fisher Scientific, 1:500), Alexa Fluor 647 (Thermo Fisher Scientific, 1:500) or DAPI 0.5ug/ml (Sigma-Aldrich). Images were processed for analysis as described below.

### Dendritic and somatic mitochondrial length, density and dendritic protrusion measurements

Hippocampal neuron cultures with a density of 2 × 10^4^ per well were seeded on Lab-Tek chamber slides (Nunc, 177445), then the neurons were infected by lentivirus expressing mitochondrial targeted Dsred at 6 DIV. At 10–13 DIV the neurons were treated with 1  μM oligomycin A for 30 min and then fixed with 4% paraformaldehyde (Sigma-Aldrich) for 30 min. After blocking with 5% BSA + 0.2% Triton X-100 (Sigma-Aldrich) for 30 min at room temperature, ActinGreen 488 ReadyProbes Reagent (Life Technologies, R37110) was used to stain F-actin to visualize dendritic protrusions. Images were collected under a Nikon confocal microscope using 40 × Oil Immersion Objective Lens at 0.5 μm thickness of each step size^14^. Dendritic segments between 70 and 100 μm from the soma were used for the analysis. The measurements of dendritic mitochondrial length, density and dendritic protrusion were conducted by using Nikon NIS Advanced Research Elements 4.13.00. A Dsred labeled particle with clear boundary was considered to be a mitochondrion. The appendages stemming from the dendrites were considered as a dendritic protrusion and they were manually classified based on the length (L), head width (dh), and neck width (dn) as previously described^56^. Mushroom spines have characteristically large heads and narrow bodies, thus are categorized as having dh/dn ≥ 1.5. Stubby spines that are characteristically short have ratios 1 ≥ dh/dn and 1 ≥ L/dn. Filopodia have characteristically long necks, can exceed the length of thin spines and are defined as having dh/dn < 1.2 and L/dn > 3. Lastly, thin spines are longer than stubby spines and have narrow heads and bodies, they are defined as ratios 1.5 > dh/dn ≥ 1 and 3 ≥ L/dn > 1. The somatic mitochondrial density, length and area were analyzed by Nikon NIS Advanced Research Elements 4.13.00. And somatic mitochondrial network was reconstructed by Fiji package of Image J (National Institutes of Health, NIH, https://imagej.nih.gov/ij/links.html).

### Statistical analysis

Statistical comparisons were performed using GraphPad Prism 8 software (https://www.graphpad.com/scientific-software/prism/). No randomization was performed. But data collection and analysis were conducted blindly to the conditions of the experiments by researchers blinded to the group performed. Two-way ANOVA followed by Bonferroni post hoc analysis or unpaired Student's t-test followed by Bonferroni post hoc analysis were applied for data analysis^26^. Numbers of replicates and *P* value are stated in each figure legend. All data were expressed as the mean ± s.e.m. Significance was indicated by the following symbols: *P* value was *P < 0.05, **P < 0.01, ***P < 0.001.

## Supplementary information

Supplementary file 1
