# Supplementary material for: Caspase inhibition rescues F1Fo ATP synthase dysfunction-mediated dendritic spine elimination
Source: Sci Rep. 2020 Oct 16;10:17589. doi: 10.1038/s41598-020-74613-9 (PMC7568535; doi:10.1038/s41598-020-74613-9)
Supplement: Supplementary file 1 [file 41598_2020_74613_MOESM1_ESM.pdf]

# Caspase inhibition rescues F1Fo ATP synthase dysfunction-mediated dendritic spine elimination

Hao Chen, Jing Tian, Lan Guo, Heng Du*

Department of Biological Sciences, The University of Texas at Dallas.800 west Campbell Rd, Richardson, TX, USA. 75080

*Corresponding Author:

Heng Du MD PhD
Associated professor
Department of Biological Sciences
The University of Texas at Dallas
Heng.du@utdallas.edu
972 883 3532

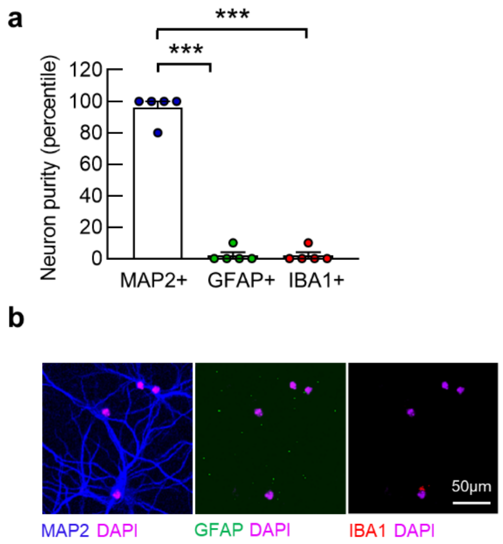


**Fig.S1. Characterization of cultured cells. (a)** Quantitative analysis of the percentages of MAP2 (Blue, a marker for neurons) positive, GFAP (Green, a marker for astrocytes) positive, IBA1 (Red, a marker for microglia) positive cells in the culture. Unpaired student's t-test; NS, not significant. *n* = 100~120 cells per group. **(b)** Representative images of MAP2 (Blue), GFAP (Green), IBA1 (Red) and DAPI (Purple) staining. Scale bar = 50μm.

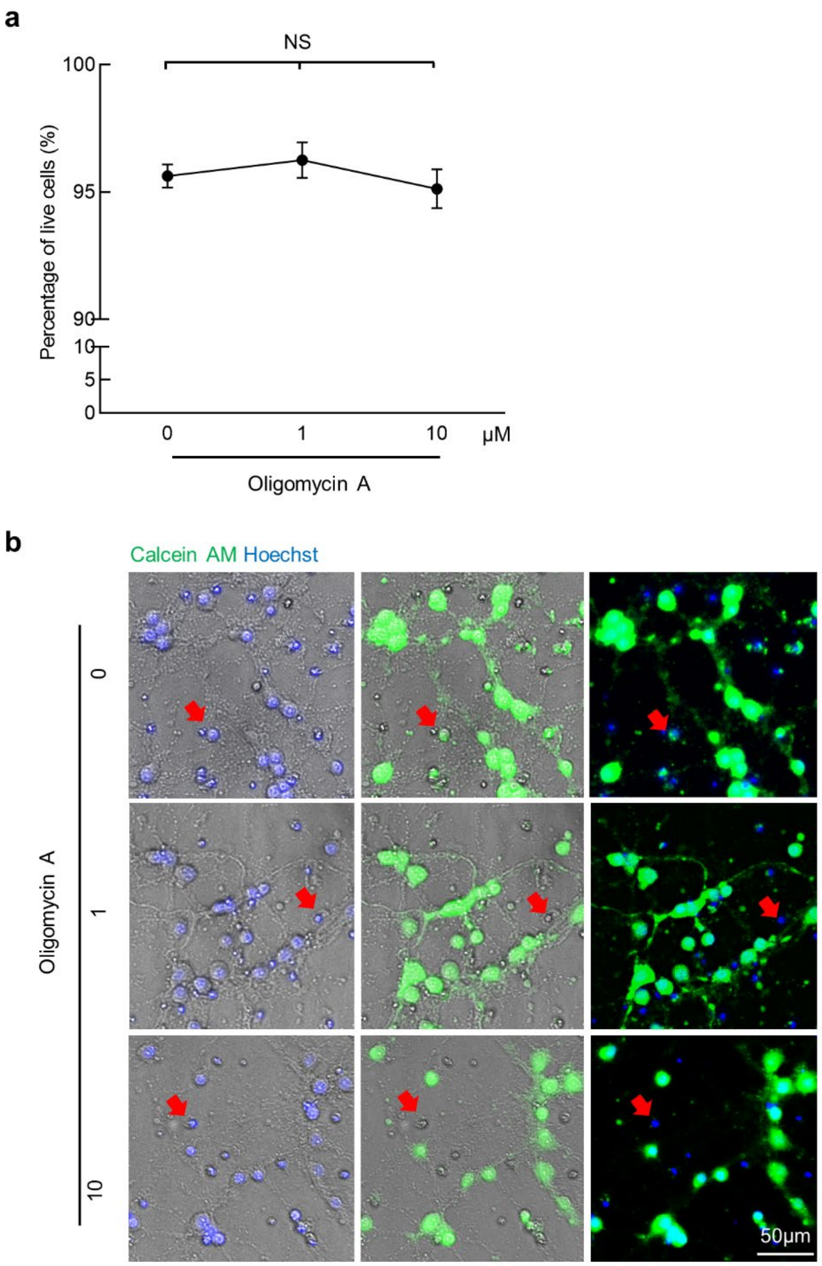


**Fig.S2. Oligomycin A treatment does not affect neuron viability. (a)** Quantitative analysis of Calcein AM (Green) positive neurons were compared under different doses of oligomycin A treatments. Unpaired student's t-test; *NS, not significant*. *n* = 250~300 cells per group. **(b)** Representative images of Calcein AM (Green) and Hoechst (Blue), red arrows indicates Calcein negative staining. Scale bar = 50μm.

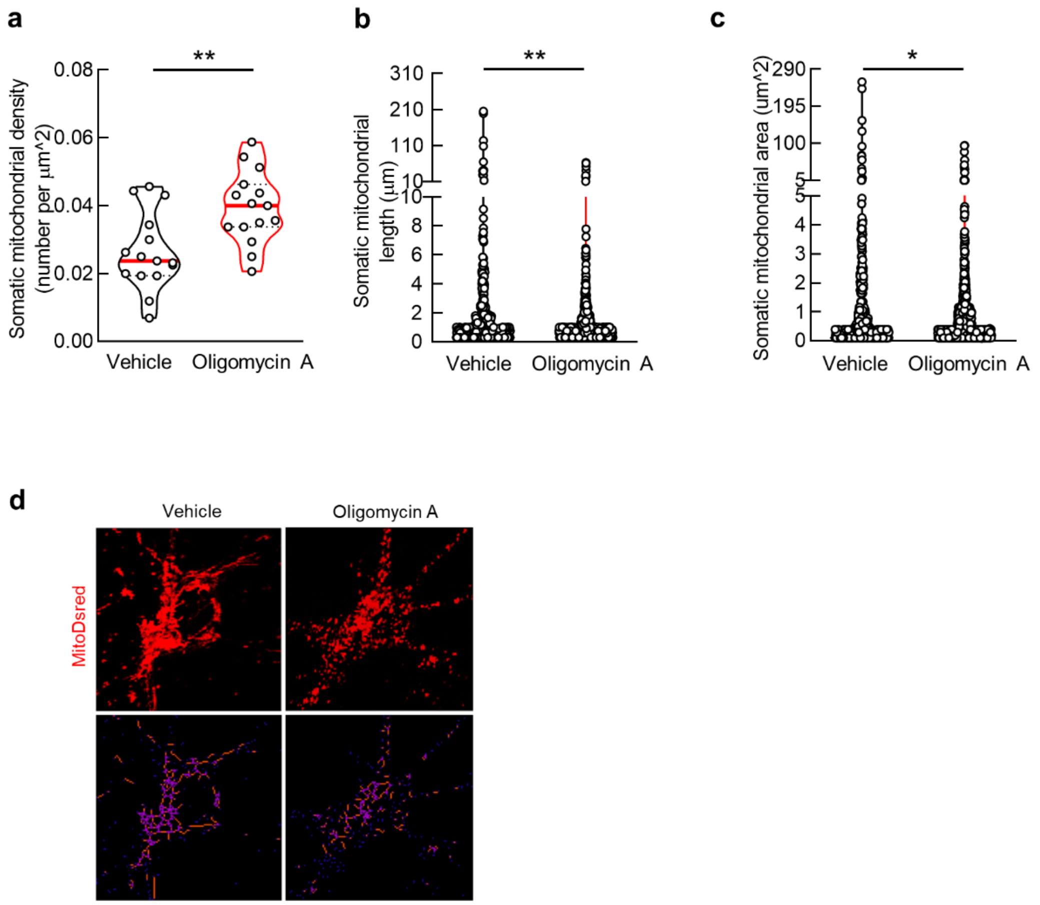


**Fig.S3. Oligomycin A treatment upregulates somatic mitochondrial fragmentation. (a)** Somatic mitochondrial density of cultured hippocampal neurons with vehicle or 1µM oligomycin A treatment. Unpaired student's *t*-test; ** *P* < 0.01. *n* = 15 neurons. **(b)** Somatic mitochondrial length of cultured hippocampal neurons with vehicle or 1µM oligomycin A treatment. Unpaired student's *t*-test; ** *P* < 0.01. *n* = 15 neurons. **(c)** Somatic mitochondrial area of cultured hippocampal neurons with vehicle or 1µM oligomycin A treatment. Unpaired student's *t*-test; ** *P* < 0.01. *n* = 196 and 208 mitochondria, respectively. **(d)** Representative images of mitochondria in the soma of hippocampal neurons, lower panels are reconstructed mitochondrial network by Image J. Scale bar = 5µm.

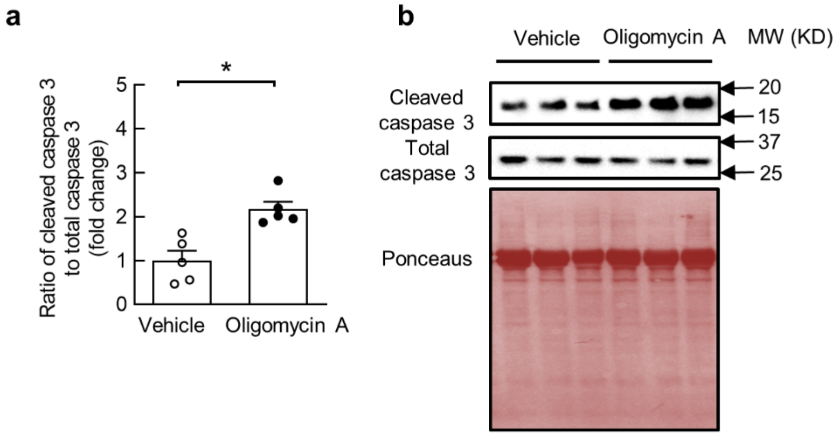


**Fig.S4. Oligomycin A treatment elevates cleavage of caspase 3 in neurons. (a)** Immunoblotting analysis of caspase 3 cleavage levels in vehicle or 1μM oligomycin A treated neurons. Unpaired student's t-test; * $P < 0.05$. $n = 5$ samples per group. **(b)** Representative bands of cleaved and total caspase 3.

a

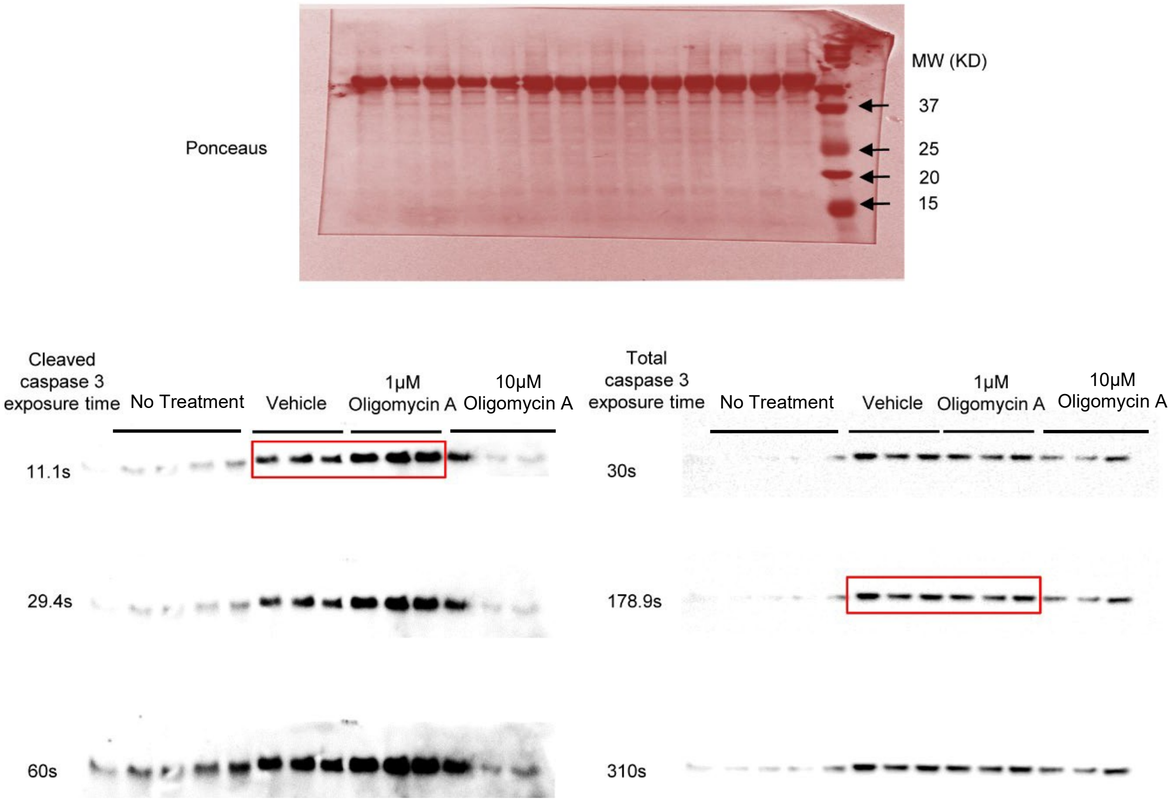


**Fig.S5. Original images of Fig.S3**. **(a)**The images are cropped from same gel, red boxes stand for where the representative images cropped from.
